# Nonfimbrial Adhesin Mutants Reveal Divergent *Escherichia coli* O157:H7 Adherence Mechanisms on Human and Cattle Epithelial Cells

**DOI:** 10.1155/2021/8868151

**Published:** 2021-01-29

**Authors:** Matthew R. Moreau, Indira T. Kudva, Robab Katani, Rebecca Cote, Lingling Li, Terrance M. Arthur, Vivek Kapur

**Affiliations:** ^1^Department of Veterinary and Biomedical Sciences, The Pennsylvania State University, University Park, PA, USA; ^2^Food Safety and Enteric Pathogens Research Unit, National Animal Disease Center, Agricultural Research Service, U.S. Department of Agriculture, Ames, IA, USA; ^3^Department of Animal Science, The Pennsylvania State University, University Park, PA, USA; ^4^The Huck Institutes of the Life Sciences, The Pennsylvania State University, University Park, PA, USA; ^5^Roman L. Hruska U.S. Mean Animal Research Center, Agricultural Research Service, U.S. Department of Agriculture, Clay Center, NE, USA

## Abstract

Shiga toxin-producing, enterohemorrhagic *Escherichia coli* (EHEC) serotype O157:H7 is a major foodborne pathogen causing symptoms ranging from simple intestinal discomfort to bloody diarrhea and life-threatening hemolytic uremic syndrome in humans. Cattle can be asymptomatically colonized by O157:H7 predominantly at the rectoanal junction (RAJ). Colonization of the RAJ is highly associated with the shedding of O157:H7 in bovine feces. Supershedding (SS) is a phenomenon that has been reported in some cattle that shed more than 10^4^ colony-forming units of O57:H7 per gram of feces, 100–1000 times more or greater than normal shedders. The unique bovine RAJ cell adherence model revealed that O157:H7 employs a LEE-independent mechanism of attachment to one of the RAJ cell types, the squamous epithelial (RSE) cells. Nine nonfimbrial adhesins were selected to determine their role in the characteristic hyperadherent phenotype of SS O157 on bovine RSE cells, in comparison with human HEp-2 cells. A number of single nucleotide polymorphisms (SNPs) were found amongst these nonfimbrial adhesins across a number of SS isolates. In human cells, deletion of *yfaL* reduced the adherence of both EDL933 and SS17. However, deletion of *eae* resulted in a significant loss of adherence in SS17 whereas deletion of *wzzB* and *iha* in EDL933 resulted in the same loss of adherence to HEp-2 cells. On RSE cells, none of these nonfimbrial deletion mutants were able to alter the adherence phenotype of SS17. In EDL933, deletion of *cah* resulted in mitigated adherence. Surprisingly, four nonfimbrial adhesin gene deletions were actually able to confer the hyperadherent phenotype on RSE cells. Overall, this study reveals that the contribution of nonfimbrial adhesins to the adherence mechanisms and functions of O157:H7 is both strain and host cell type dependent as well as indicates a possible role of these nonfimbrial adhesins in the SS phenotype exhibited on RSE cells.

## 1. Introduction

Shiga toxin-producing *Escherichia coli* are the most prevalent of the 6 major pathotypes of *E. coli* and are responsible for approximately 265,000 infections annually, in the US alone [1, 2]. The serotype O157:H7, an enterohemorrhagic *E. coli* (EHEC), is the cause of 40% of those infections whose clinical symptoms range from watery or bloody stools and abdominal cramps to renal damage and/or failure and potentially life-threatening hemolytic uremic syndrome (HUS) [2–4]. Most humans are infected by O157:H7 through consumption of meat or leafy vegetables contaminated with bovine fecal matter [3, 5].

Despite causing disease in the human host, cattle colonized with O157:H7 are asymptomatic [1–3]. Cattle are the primary reservoirs and the primary site of colonization for O157:H7 in cattle is the rectoanal junction also known as the RAJ [2]. This special location at the terminal end of the gastrointestinal tract (GIT) is comprised of two major cell types, the follicle-associated epithelia (FAE) and the rectoanal junction squamous epithelia (RSE) [6–8]. The typical shedding rate of O157:H7 from the RAJ is 10–100 colony-forming units per gram (CFU/g) of feces [8]. Supershedder (SS) cattle, on the other hand, shed ≥10^4^ CFU/g of O157:H7 [9]. Mathematical modeling of SS cattle revealed they are responsible for greater than 96% of O157:H7 found in the environment despite the fact that the SS cattle make up less than 10% of a cattle herd [9–11]. Another study in Scotland showed that 80% of all transmission events to other cattle on farms were caused by SS *E. coli* [10, 12].

Approximately 300 CFU to potentially 10^7^ CFU of O157:H7 is required for colonization of calves and cattle whereas only 10–100 CFU of O157:H7 is required to infect humans [13, 14]. Because SS cattle shed two to three orders of magnitude more than the infectious dose for both humans and cattle per gram of feces, they are an important driver of the overall epidemiology of O157:H7 to both hosts. Much about the SS phenomenon is unknown, but is likely to center around three main parts: the host, the bacteria, and the environment, all of which can have a significant role in this phenotype [9, 11]. There have been many studies that have shown that the strain relationship among SS cattle and various other conditions (weather, time of day, and time of year) all can contribute to changes in the shed rate of O157:H7 [15–18]. Given that these factors play critical roles in shedding phenotype, it is possible that all cattle could have the potential to be a supershedder, furthering our need to understand the mechanisms that drive this process.

To colonize a host, any bacteria need to find a niche where they can compete for resources and be able to anchor themselves into that niche in order to not be stripped away through movement through the area. In order to attach to host cells, most pathogens express adhesins to initiate contact with surfaces (such as eukaryotic cell membranes or extracellular matrix) to confer adherence to these surfaces and allow for the synthesis of other adherence molecules. The locus of enterocyte effacement (LEE) is the intimate attachment locus that encodes the adhesin, intimin, and its cognate receptor which is translocated into and expressed by the host cell, Tir [19]. Attachment is mediated through type III secretion system (T3SS) encoded for by the operon, and its effectors. All of these factors create the pedestal formation associated with O157:H7 LEE-driven adherence as well as modulate immune signaling, actin polymerization, and other eukaryotic cellular pathways [2, 19, 20].

After adherence, O157:H7 produces toxins (such as Shiga toxin and hemolysin toxin) to cause damage to the endothelial and epithelial cells, which release key nutrients the bacteria requires to survive [21–23]. Shiga toxin is the reason most of the patients that have an O157:H7 infection cannot be treated by routine antibiotics. Shiga toxin is expressed by the bacteria at a basal level as a virulence factor, but treatment of the infection with antibiotics can stimulate activation and release of the Shiga toxin-containing phage and with it an increased synthesis and release of Shiga toxin. Once into the bloodstream, Shiga toxin binds the Gb3 receptors primarily expressed by the endothelial cells and more specifically in the kidney [24]. Interestingly, that is why cattle do not show any signs of illness when infected with O157:H7; they do not have the Gb3 receptor required for Shiga toxin to affect cells [25]. Despite showing no major pathophysiological signs of disease, one question that remains unresolved is why O157:H7 can persist in cattle for an extended period. Recent evidence from a variety of studies suggests that Shiga toxin alleles can bind to CD77 which is involved in the mitogen-activation response. Most cattle cells, including epithelial cells and leukocytes, have this receptor expressed at various stages of development and differentiation. At high density, this receptor can trigger the MAP pathway-mediated apoptosis, especially for fully differentiated crypt cells in cattle, which may affect shed rate [26]. If the Shiga toxin is taken up by another cell type through a non-Gb3-mediated endocytosis, Shiga toxin has been shown to reduce or mostly prevent that cell from producing functional nuclear factor of kappa B, NF*κ*B [24].

Aside from the importance of factors such as the LEE operon and Shiga toxins, O157:H7 must be able to adhere to the apical cell surface through what is most likely ligand-receptor binding conferred by other encoded adhesins. For many bacteria, adhesins are placed into two distinct categories: fimbrial and nonfimbrial. Fimbrial adhesins are long, polar, fiber-like extensions which are terminated by a receptor-specific adhesin [27]. Nonfimbrial adhesins are generally single subunit proteins that are similar to type V secretion systems also known as autotransporters. Most autotransporters contain three critical domains: the *β*-barrel that anchors the protein into the bacterial membrane, the linker domain that is the flex arm of the effector domain which usually contains an adhesin or effector that is autotransported through the *β*-barrel and interacts with host cell proteins [28, 29]. Many O157:H7 isolates including EDL933 and supershedder strains such as SS17 contain many different loci that have both fimbrial and nonfimbrial adhesins [19, 30].

Many of the adhesins found in O157:H7 have been shown to be critical for adherence. The *cah* protein (calcium binding antigen 43 homolog) was previously shown to be important for *E. coli* cell aggregation and adherence to human cells [31, 32]. The Iha protein serves a dual purpose to the bacterium as a transporter of siderophores such as enterochelin bound to iron, as well as a Fur-regulated adhesin [33, 34]. OmpA has also been shown to be important for adherence on a number of different surfaces including plants and animals [35, 36]. Two final examples of nonfimbrial autotransporter adhesins are *yeeJ* and *yfaL* which enhance O157:H7 binding to eukaryotic cells [29, 37]. There are many other adhesins that are expressed by some EHEC that have been shown to be adhesins and others hypothesized to be such based on sequence homology to other adhesins, such as EaeH in enterotoxigenic *E. coli* [38, 39].

The underlying difference in adherence mechanisms employed by EHEC, in particular O157:H7, to adhere to RSE cells has not been defined, nor has the adhesins used prior to LEE activation on human epithelia been identified. The biggest reason for this is the functional redundancy that O157:H7 has with regard to its adhesins. It has been previously reported that SS isolate SS17 displays a LEE-independent, strong, and aggregative adherence phenotype on RSE cells but only diffuse and moderate adherence to HEp-2 cells [40, 41]. A separate study revealed that the SS isolates do not share extensive genetic similarities as defined by PFGE relatedness [9].

We previously have a fully sequenced SS isolate (SS17, Accession number CP008805) and its genome was analyzed in order to identify genetic differences compared to the previously well-characterized O157:H7 strains through reverse genetic approaches [41]. In this study, we have sought to determine the genes involved in the adherence phenotype previously attributed to SS17 and performed comparative adherence profiling both quantitatively and qualitatively comparing O157:H7 isolates SS17 and EDL933 (as a reference). These studies provide evidence that the adherence mechanisms employed by SS and non-SS O157:H7 isolates to HEp-2 cells are different. They also suggest the initial adherence of O157:H7 strains onto bovine RSE cells is different than that of O157:H7 strains to human epithelial cells, providing evidence that the initial adherence of O157:H7 strains to human and bovine epithelial cells requires different adhesins. Lastly, these analyses suggest an intricate and complex regulatory network that is involved in the difference in the adherence phenotypes of O157:H7 strains to RSE cells.

## 2. Materials and Methods

### 2.1. Bacterial Strains

SS17 and all supershedder isolates of O157 (SS1, SS7, SS12, SS27, SS42 SS52, SS67, SS77, SS131, RM11333, and RM11326) were initially obtained and characterized by the USDA Meat Animal Research Center, Clay Center, NE. RM11333 and RM11326 were environmental isolates that were discovered to have high biofilm potential. Isolates were collected and characterized as previously described [9]. EDL933 isolate was obtained from Edward Dudley, Pennsylvania State University. EDL933 was originally obtained from samples of a 1982 hamburger outbreak of O157:H7 in Michigan [42].

### 2.2. Determining Adhesins of Interest

**Figure 1:**
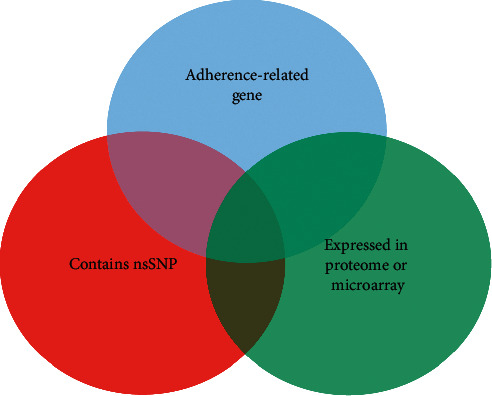
Gene selection process. The whole genome of SS17 was fully annotated through RAST and manual annotations [5]. Adherence genes were compared against a previously performed proteome and comparison of the genes that contained nsSNPs compared to a non-SS isolate EDL933. Any nonfimbrial adhesins matching two of these three criteria were considered.

The gene selection process, as shown in Figure 1, was performed by initially cross-referencing genes encoding adhesins in O157:H7 to genes that have acquired nonsynonymous single-nucleotide polymorphisms (nsSNPs) in SS17 compared to EDL933. These selected genes were then compared to a proteome analysis provided by Kudva and colleagues on EDL933 adherence to RSE cells [40]. Nonfimbrial genes that shared at least two of the three criteria were selected as genes of interest (GOI) and used for subsequent phylogenetic and gene knockout analyses.

### 2.3. Phylogenetic Analysis Based on SS Nonfimbrial Adhesin SNPs

In brief, glycerol stocks from the original stabs of the 12 SS isolates were struck out onto LB agar and isogenic colonies were picked and grown overnight in LB, and DNA was isolated using the DNeasy Blood and Tissue Kit (Qiagen, Valencia CA) and eluted in ddH_2_O. From the DNA prep, 2 *µ*l was added to a 20 *µ*l PCR with the newly constructed S1 and S2 primers (Table S1) that annealed to sequences approximately 250 bp flanking the nsSNP(s). Products were run out on a 1% agarose gel (1/10 volume) and the remaining product was cleaned by filter exclusion and sent for sequencing in both the forward and reverse directions. Sequences were aligned using the ApE Plasmid Editor and SeqMan (DNASTAR, Madison WI; Lasergene v. 10) programs and analyzed for either the conservation of a particular nsSNP or the same gene with a different nsSNP local to the original nsSNP by alignment to EDL933 and SS17 for references.

Phylogenetic analysis was performed using MEGA5 [43]. Codons containing the SNPs in 9 genes were aligned by the formation of FASTA format to generate MEGA alignment. The deletion in *cah* was accounted for by a coded change in a codon. The alignment of the 27 nucleotides was then used to generate the phylogenetic through a bootstrap 1000 maximum nucleotide likelihood of all the SS isolates. Confidence percentages and sequence alignments were added to the phylogenetic tree to indicate the sequence/SNP tested linked to a certain gene and certain isolate.

### 2.4. Gene Knockouts by Single-Step Inactivation

Bacterial genes were knocked out by single-step inactivation as previously described [44]. In brief, primers were designed for the genes of interest that flocked the target sequence in orientation for homologous recombination (Table S2). The inner part of the primer was designed with homology to the *kanR* cassette on pACYC177. Next, pKD119 (pBAD-*λ*RED) was electroporated into both EDL933 and SS17; linear *kanR* cassettes were also electroporated after induction of the *λ*RED by glucose during growth and recovery [41]. The *kanR* cassette recombined into the chromosome was selected for by plating on LB + Kan50 (50 *µ*g/mL) followed by colony PCR by internal and external primers (found in Table S3) and 1% agarose gel analysis of the bands. PCR results were then sent for sequencing at the Pennsylvania State University Sequencing core using the primers found in Table S3. For SS17, *eae* was also chosen as a LEE-mediated adherence control (as previous data had been obtained for EDL933 with pooled antibodies against LEE and *eae* mutants for both RSE and HEp-2 adherence; [40]).

### 2.5. RSE Cell Adherence Assay

The RSE cell adherence assay was performed as previously described [40, 41]. Briefly, EDL933 and SS17 (with their full complement of mutants) were grown overnight in Dulbecco modified Eagle medium-low glucose (DMEM-LG; Invitrogen, Grand Island, NY) at 37°C without shaking. Bacteria were pelleted, resuspended in saline, and added to 1 mL DMEM containing 10^5^ RSE cells/mL at a ratio of 10 : 1. Cells and bacteria were incubated for 4 hours with shaking at 37°C prior to being processed and placed on polysine slides (Thermo Scientific, Rockford, IL) as described previously [40, 41]. Fixed slides were stained with 1% toluidine blue or fluorescent antibodies for O157 and RSE-cytokeratins [40, 41]. The adherence patterns were qualitatively discerned by light or fluorescence microscopy, and quantitative scoring was performed by counting the number of bacteria per cell in two separate blind trials of 4 technical replicates each [40, 41]. The adherence pattern and efficiency of each mutant were subsequently compared back to its parent strain.

### 2.6. HEp-2 Adherence Assay

HEp-2 adherence assay was performed as described previously [40]. In short, O157:H7 isolates (SS17 and EDL933) and their mutants were added to HEp-2 cells in chamber slides at a ratio of 10 : 1. Postassay, the slides were stained with 1% toluidine blue and visualized at 40x magnification on a slide to qualitatively and quantitatively discern adherence pattern and efficiency of each mutant tested back to its parent strain.

### 2.7. Statistical Analysis

Statistical analysis of adherence assays was performed using Microsoft Excel and a two-tailed *t*-test of raw data averages compared to each mutant. Statistically significant differences were calculated for *p* values <0.05.

## 3. Results

### 3.1. Gene Selection and Phylogenetic Analysis of SS Isolates Based on Nonfimbrial Adhesins

Nine nonfimbrial genes involved in adherence were chosen from the criteria shown in Figure 1. The criteria considered for the genes of interest were that the gene and/or gene product must be related to adherence, exhibit a nonsynonymous single-nucleotide polymorphism (nsSNP) in SS17 compared to EDL933, and/or expressed in the proteome array performed by Kudva and colleagues. Two of the genes, *iha* and *ompA*, had no SNPs in SS17 compared to EDL933. The genes *eivA*, *csgG*, *wzzB*, and *eaeH* all had one nsSNP compared with EDL933 and the *yfaL* gene had 3 nsSNPs. Sequencing of the intimin gene (*eae*) revealed no mutations, which was expected as important as the gene is in conferring intimate adherence to human epithelia. The last gene *cah* encoded for a nonsense mutation that deleted the final ∼850 base pairs of the gene. In order to confirm the previous observation that intimin is not involved in the adherence of O157:H7 to RSE cells, we knocked out the intimin gene, eae [40, 41].  Table 1 shows all of the genes selected, the changes in amino acids as a result of SNPs in the genes, and function of the genes of interest.

**Figure 2:**
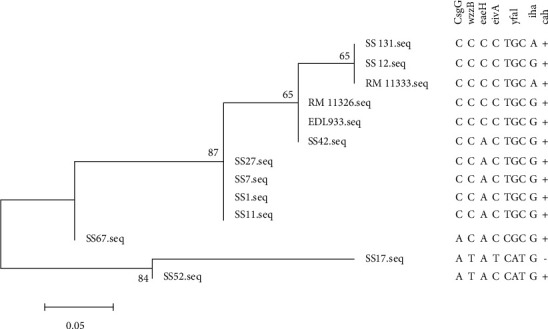
Phylogenetic analysis of SS isolates to EDL933 based on adhesins. A maximum likelihood bootstrap analysis was performed using the adhesion-related genes that displayed SNPs compared to the EDL933 isolate. There were 11 other isolates in combination with SS17 and EDL933 run in this analysis. This analysis revealed that, solely based on these adhesins, the isolates clustered close to their lineages and PFGE types. The panel to the right is aligned with the nucleotide changes in each gene aligned to the nucleotides found in the corresponding isolate. The scale (0.05) indicates distance based on the number of changes/sites.

The sequences of all the adhesins selected were obtained for 11 other SS isolates. These sequences were first used to identify if any of the SNPs found in SS17 were also found in other SS isolates, where none were completely conserved (Table 2). Using a maximum likelihood of 27 sites (nine codons) across all of the genes selected, a phylogenetic map was created (Figure 2). As Figure 2 demonstrates, there is a clear clustering of SS isolates that is correlative to their lineages and PFGE types previously determined.

### 3.2. Gene Deletion Confirmations

**Figure 3:**
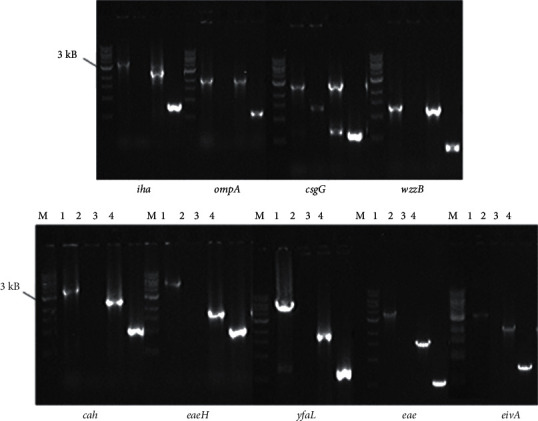
Gel electrophoretic confirmation of deletions. Above is an agarose gel picture of colony PCR products obtained after single-step inactivation of each gene. Each lane marked is as follows: M-1 kb marker (ladder), 1&2 SS17 parental strain with primers A&B and A&C, respectively, and 3&4 SS17 deletion mutants with primers A&B and A&C, respectively. Primers indicated are the primers in Table S3. Primers A&B are specific to the gene indicated under each gel image and primer C is the matching reverse primer specific to the Kan cassette that is partnered with each gene's primer A.

In order to delete genes, the *kanR* cassette from pACYC177 was PCR-amplified and flanked by sequences homologous to the gene being targeted, matching the orientation of the gene. Using internal and external primers to the *kanR* cassette and the neighboring sequences of the target gene, respectively (Table S3), colony PCR was performed to confirm the mutation. The successful mutations of these genes in both SS17 and EDL933 were confirmed by gel electrophoresis (Figure 3), and mutant strains were subsequently used in the adherence studies with RSE and HEp-2 cells.

### 3.3. SS17 and EDL933 Adhesin Mutants on HEp-2 Cells

**Figure 4:**
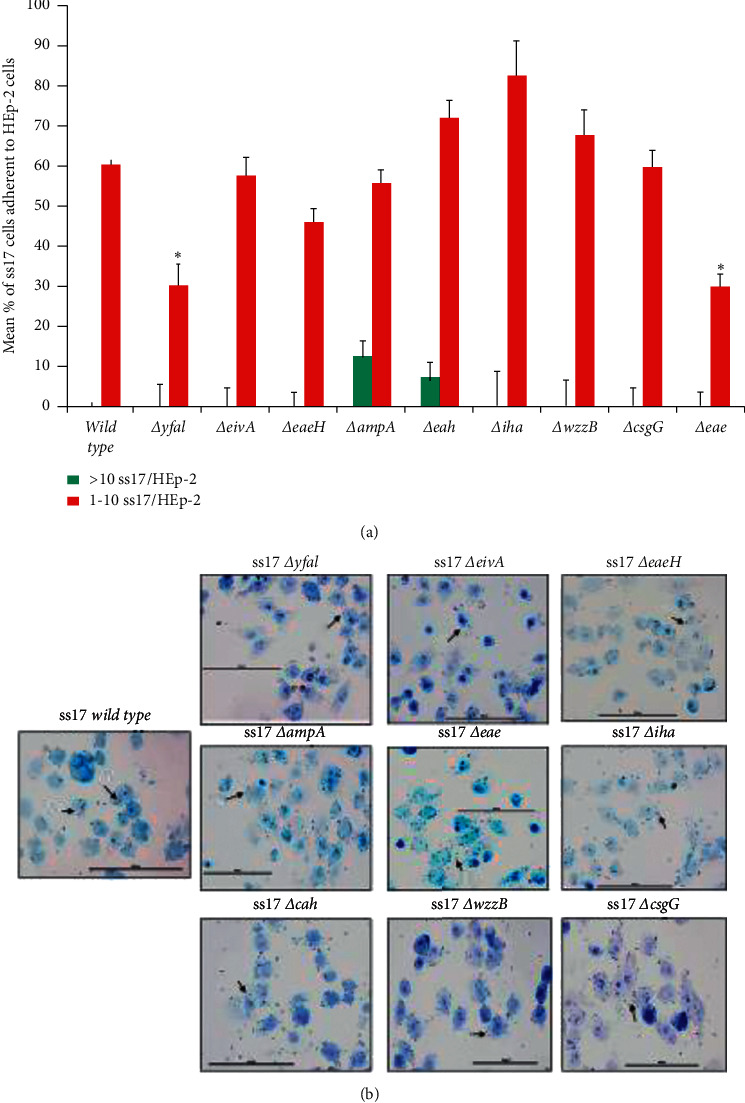
SS17 adherence mutant phenotypes on HEp-2 cells. Adherence of the parental strain and subsequent adherence-associated gene mutants of SS17 were tested for adherence quantitatively (a) and qualitatively (b). As the results show, SS17Δ*yfal*, Δ*eaeH*, and Δ*eae* are all deficient in adhering to HEp-2 cells compared to the parent SS17 strain. (a) Each bar is representative of four technical replicates per slide where 20 cells/spot were counted over two trials. The bar is the mean percentage of adherent bacteria (>10 or 1–10) per HEp-2 cell over the two trials. (b) Toluidine blue-stained slides are used to see the “adherence morphology” of SS17 to HEp-2 cells. The scale bar for each panel is set to 100 *μ*m. Asterisks denote *p* value <0.05.

**Figure 5:**
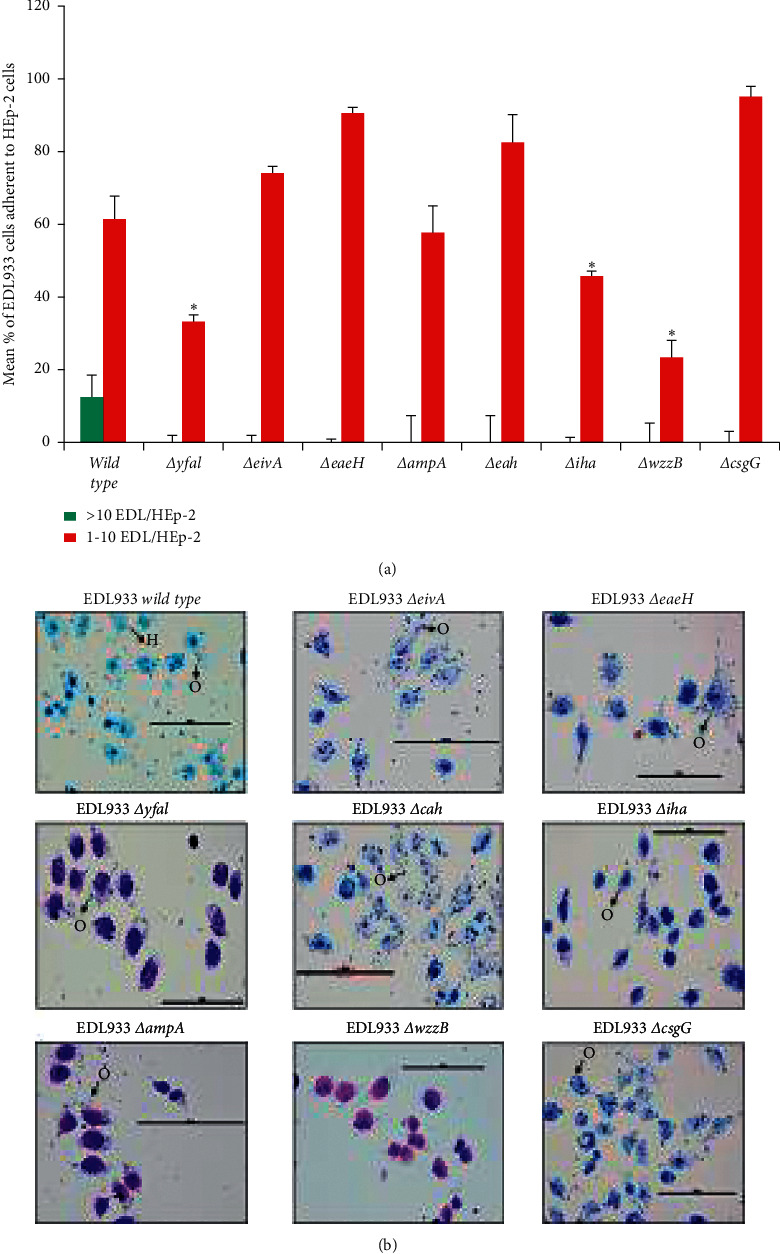
EDL933 adherence mutant phenotypes on HEp-2 cells. Adherence of the parental strain and subsequent adherence-associated gene mutants of EDL933 were tested for adherence quantitatively (a) and qualitatively (b). Both indicate that EDL933Δ*yfaL*, Δ*iha*, and Δ*wzzB* are all deficient in adhering to the HEp-2 cells compared to wild-type EDL933. (a) Each bar is representative of four technical replicates per slide where 20 cells/spot were counted over two trials. The bar is the mean percentage of adherent bacteria (>10 or 1–10) per HEp-2 cell over the two trials. (b) Toluidine blue-stained slides are used to see the “adherence morphology” of EDL933 to HEp-2 cells. The scale bar for each panel is set to 100 *μ*m. Asterisks denote *p* value <0.05.

The bacterial adherence pattern of SS17 on HEp-2 cells was diffuse and moderate, which was in stark contrast to the aggregative and strongly adherent phenotype exhibited on RSE cells. Not surprisingly, *eae* (intimin) deletion showed lack of adherence to HEp-2 cells (Figure 4). Two other mutations, SS17ΔyfaL and SS17Δ*eaeH*, also inhibited the ability of SS17 to adhere to the HEp-2 cells both quantitatively (Figure 4(a) and Table S4) and qualitatively (Figure 4(b)). As Figure 5 shows, EDL933 had the same adherence pattern on HEp-2 cells as SS17 (diffuse and moderate) and antibodies against LEE have previously demonstrated a severe reduction in adherence [40]. As seen in Figure 4, EDL933Δ*yfaL* displayed a nonadherent phenotype, but EDL933Δ*eaeH* did not disrupt the ability of EDL933 to adhere to the HEp-2 cells. However, EDL933Δ*iha* and EDL933Δ*wzzB* both displayed nonadherent phenotypes on HEp-2 cells (Figure 5 and Table S5). None of the remaining adhesins tested showed any difference in the ability of SS17 or EDL933 to adhere to the HEp-2 cells.

### 3.4. SS17 and EDL933 Adhesin Mutants on RSE Cells

**Figure 6:**
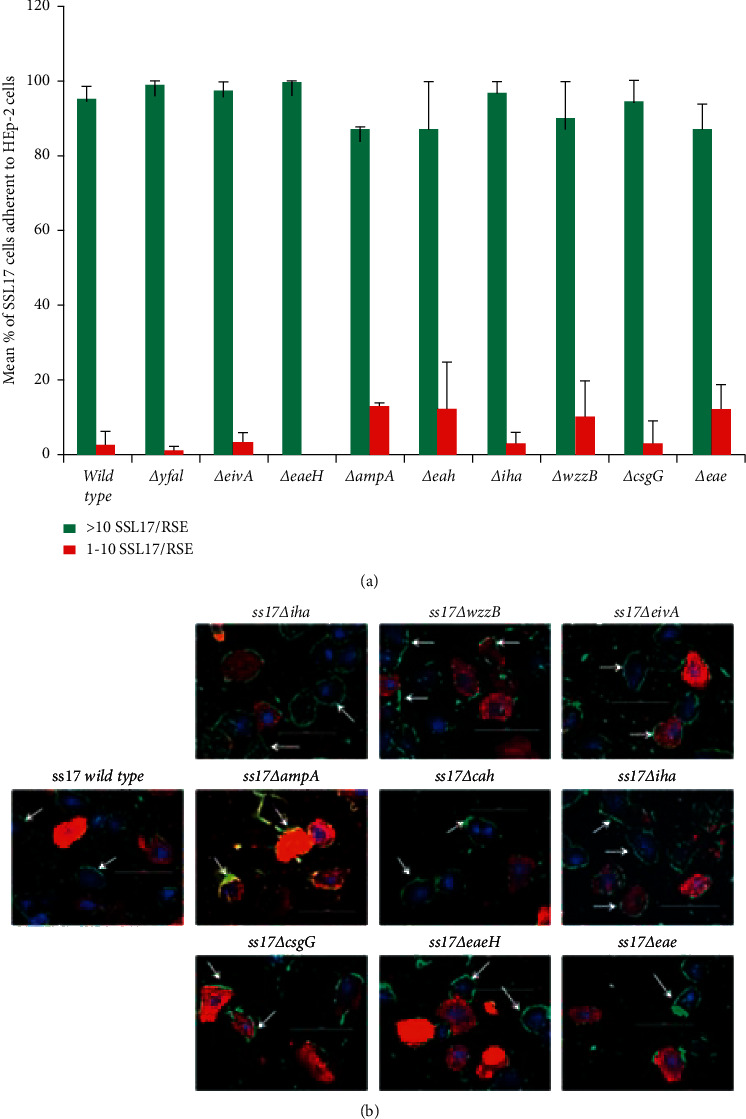
SS17 adherence mutant phenotypes on RSE cells. The parental strain and the adherence-associated gene mutants of SS17 were tested for adherence quantitatively (a) and qualitatively (b) on RSE cells. As the results show, none of the mutations significantly altered SS17's ability to bind RSE cells with its distinct aggregative, strong adherence. (a) Each bar is representative of four technical replicates per slide where 20 cells/spot were counted over two trials. The bar is the mean percentage of adherent bacteria (>10 or 1–10) per RSE cell over the two trials. (b) Immunofluorescence of SS17 adherence pattern on RSE cells. Bacteria are labeled with green, RSE cell cytokeratins with red, and RSE cell nuclei with DAPI (blue) fluorescent staining. The scale bar for each panel is set to 100 *μ*m and the white arrows show the O157 on RSE cells.

Wild-type SS17 maintained the aggregative and strong adherence pattern as approximately 95% of RSE cells sampled had greater than 10 bacteria per cell (Figure 6 and Table S4). From the nine nonfimbrial adhesin mutants in SS17 tested, none showed any change in adherence efficiency (Figure 6(a)) or adherence pattern (Figure 6(b)) compared to the wild type. As Figure 6 shows, all gene knockouts displayed no significant difference in the number of RSE cells with greater than 10 bacteria per cell (Table S4).

**Figure 7:**
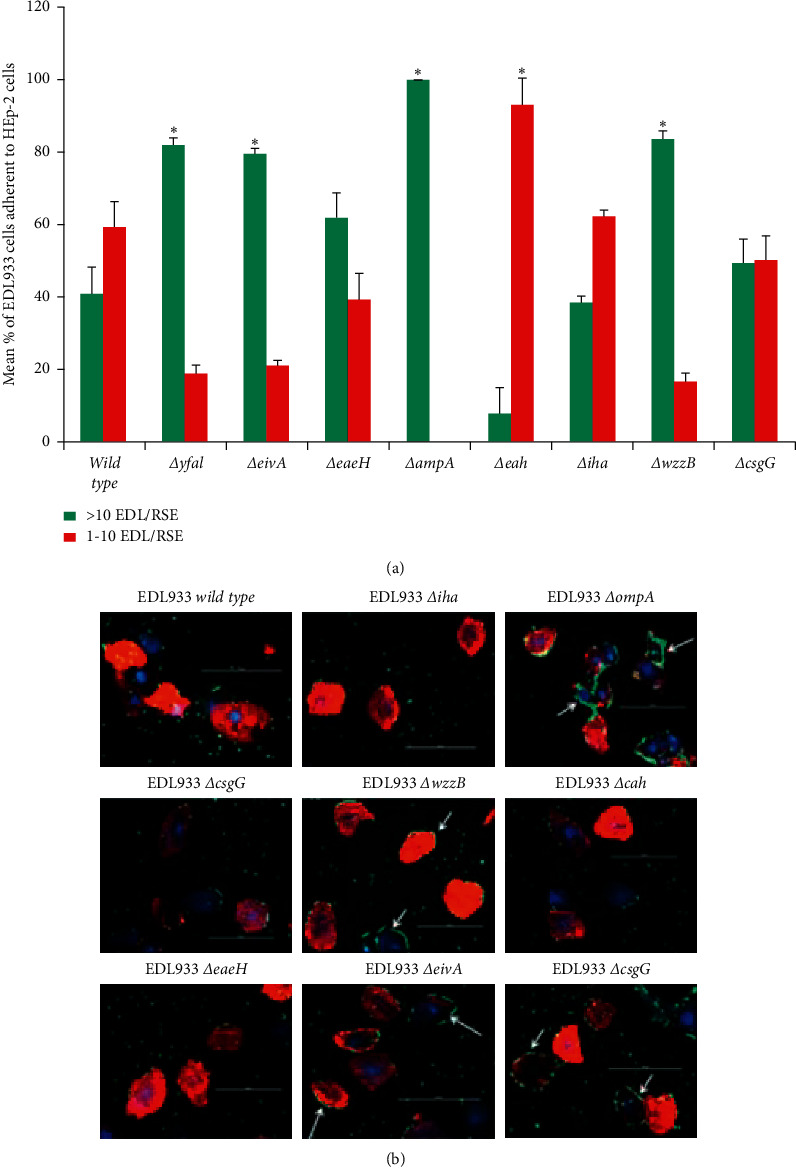
EDL933 adherence mutant phenotypes on RSE cells. The parental strain and the adherence-associated gene mutants of EDL933 were tested for adherence quantitatively (a) and qualitatively (b) on RSE cells. As the results show, the mutants of EDL933 have a lot of adherence differences compared to the parental strain. These differences are loss of adherence (EDL933Δ*iha* and Δ*cah*) and a phenotypic switch from aggregative moderate adherence to the same aggregative strong adherence exhibited by EDL933 on RSE cells (Δ*yfaL*, Δ*wzzB*, Δ*ompA*, and Δ*eivA*). (a) Each bar is representative of four technical replicates per slide where 20 cells/spot were counted over two trials. The bar is the mean percentage of adherent bacteria (>10 or 1–10) per RSE cell over the two trials. (b) Immunofluorescence of EDL933 adherence pattern on RSE cells. Bacteria are labeled with green, RSE cell cytokeratins with red, and RSE cell nuclei with DAPI (blue) fluorescent staining. The scale bar for each panel is set to 100 *μ*m and the white arrows show the O157 on RSE cells. Black asterisks denote *p* value <0.05 for gain of adherence and red for loss of adherence.

With wild-type EDL933, approximately half of the RSE cells had greater than 10 bacteria per cell and similar amount of RSE cells had 1–10 bacterial per cell (Figure 7(a)), and an aggregative, moderate adherence phenotype was observed (Figure 7(b)). EDL933Δ*cah* and EDL933Δ*iha* became less efficient at binding to RSE cells with more RSE cells having 1–10 bacteria per cell (Δ*cah*) or overall loss of adherence (Δ*iha*). Interestingly, EDL933Δ*yfal*, EDL933Δ*eivA*, EDL933Δ*ompA*, and EDL933Δ*wzzB* all showed a change in the wild-type EDL933 adherence pattern from aggregative moderate to aggregative strong, the same phenotype exhibited by the SS17 isolate (Figure 7(b) and Table S5). As Figure 7(a) shows, these results were consistent with the percentages of RSE cells with >10 EDL933 cells per RSE cell as seen with SS17.

## 4. Discussion

Enterohemorrhagic *Escherichia coli*, and serotype O157:H7 in particular, remains a primary concern in foodborne illness since many efforts to stymie its transmission have been unsuccessful [13]. With asymptomatic cattle being the primary reservoirs of O157:H7, supershedding cattle have the most influence on bacterial burden on the environment [12]. The most efficient way to prevent illness from ever occurring in humans is to prevent the colonization and spread of O157:H7 through its source [41]. The ability to colonize the RAJ is critically linked to the ability of O157:H7 to persist in cattle, and hence, this colonization event seems to be important in influencing changes in O157:H7 shedding, such as supershedding [8, 11].

Previous analysis of the RAJ revealed that this area is made up of two morphologically distinct cell populations—the follicle-associated epithelia (FAE) and the rectoanal junction squamous epithelia (RSE); both of which have been shown to interact with O157:H7 [2, 6, 40]. Taken together, the data suggest that understanding the complex nature of O157:H7 interactions with the RAJ epithelia may help elucidate conditions required for the SS adherence phenotype. To further understand the microbial components involved in adherence and/or the SS adherence phenotype as a whole, we performed a genomic and phenotypic analysis of one representative SS isolate, SS17 [40, 41]. The analysis revealed over 60 virulence genes that could be involved in the hyperadherent phenotype SS17 exhibited on RSE cells [40, 41]. Despite the conservation of this phenotype amongst all SS isolates, a previous study had shown that there is no one genetic make-up through various typing methods that could separate SS isolates from non-SS isolates as the study showed that the SS isolates alone display great genomic heterogeneity [9]. That study focused on nsSNPs as the difference in adherence efficiency and the adherence pattern of SS17 on RSE cells was distinctly different from that of EDL933 [41]. Comparative genomics between SSS17 and EDL933 revealed 4847 SNPs comparing SS17 to EDL933, suggesting that there is a multitude of genetic change that could potentially play a significant role in the adherence pattern that was previously reported, not specifically only adhesins, as our sequence data indicated in Table 2 and Figure 2 [40, 41].

The same study also showed (as does this one) that the adherence of O157:H7 to RSE cells was independent of the LEE operon [40, 41]. Both SS17Δ*eae* and SS17, in the presence of pooled anti-LEE sera, were able to maintain the SS adherence phenotype to RSE cells [40, 41], suggesting that microbial factors involved in this adherence must include non-LEE-regulated adhesins or other factors. In this study, these adhesins were targeted to attempt to find the factors and the mechanisms underlying the differences in adherence of SS17 to RSE cells compared to EDL933. By combining data on genes meeting three criteria (encoding a LEE-independent adhesin, containing an nsSNP and being part of a previously defined proteome), genes were selected for deletion. Nine adhesins or adherence-related genes that fit these criteria and covered most of the non-LEE-regulated nonfimbrial adhesins [19, 30] were selected for further analysis (Table 1).

Most microbes display genomic coevolution with their hosts, and as O157:H7 can infect both cattle and humans, it would be expected that a divergence of microbial factors is involved in the colonization of each host (as the host microenvironments are different) to maintain fitness; however, no work to our knowledge has been done to show this [45, 46]. Similarly, there has been a divergence in the ability and phenotype of SS O157:H7 isolates to adhere to RSE cells, but what factors drive this change and how they could affect the isolate's ability to attach to human cells is not well understood.

Both SS17 and EDL933 showed that adherence to HEp-2 cells, by both pattern and efficiency, was relatively the same between the two nonmutated isolates, despite the large number of SNPs between the two (Figures 4 and 5). However, the analysis of the adhesin deletion mutations revealed that the underlying mechanism of adhesion and aggregation is both strain and host cell dependent. Given the genetic heterogeneity of the SS isolates sequenced for the initial analysis of this study and the divergence of these strains from a classical strain (EDL933), this may be a function of fitness mutations and regulatory functions at the time of the interface between O157:H7 and a host. As Figure 4 shows, EDL933Δ*iha* and EDL933Δ*wzzB* displayed nonadherent phenotypes on HEp-2 cells whereas SS17Δ*eaeH* (Figure 5) displayed the nonadherent phenotype on HEp-2 cells. The remainder and corresponding mutants of both EDL933 and SS17 did not change the adherence of the other isolates. Both *wzzB* and *eaeH* contain nsSNPs between EDL933 and SS17, and it would appear that these genes and their SNPs may play a role in this divergence in adherence mechanisms to HEp-2 cells. It is possible that *wzzB* SNPs cause a change in the formation of the LPS in SS17, thereby allowing SS17 adhesins to access host cell receptors without interference. Together, these observations suggest that EDL933 and SS17 have two different underlying mechanisms of initial adherence to HEp-2 cells as well as that *wzzB*, *eaeH*, and *iha* all play a critical role in O157:H7 adherence to human epithelial cells, but their contributions to that mechanism may be strain dependent.

Figures 4 and 5 show that the Δ*yfaL* mutation in both isolates decreased adherence to HEp-2 cells, suggesting the nsSNPs in *yfaL* does not affect the ability of O157:H7 isolates to adhere to human epithelial cells or influence the overall mechanism. This also suggests that YfaL is an integral part of a conserved adherence mechanism of O157:H7 to human epithelia. To our knowledge, this is the first time that *yfaL* and *eaeH* have been shown to be critical for the adherence of O157:H7 to human epithelial cells. Taken together, these data suggest that the ability to adhere to human epithelial cells involves central factors (such as *yfaL*), but the maintenance of that adherence would depend on different factors that could be strain specific. These data also suggest that the nsSNPs in *eaeH* and *wzzB* may change the overall function or regulatory mechanisms involved with these genes to cause them to be more important for the ability of certain strains to adhere to human epithelial cells.

Despite the fact that SS17Δ*eaeH* showed a nonadherent phenotype on HEp-2 cells, the same mutation did not confer a loss of or even a change in adherence of SS17 to RSE cells. In fact, none of the mutations could cause a change in adherence of SS17 to RSE cells as all mutants were able to maintain the strong, aggregative adherence where over 85% of the RSE cells had >10 O157:H7 per cell. The previous work that showed the difference in adherence phenotypes between SS17 adherence on HEp-2 cells (diffuse and moderate) vs. RSE cells (strong and aggregative) would have suggested a change in the overall adherence mechanism, but here we show that there actually is a difference in the factors involved [41]. We can conclude that none of the nonfimbrial adhesins chosen for this study are singly responsible for the hyperadherent phenotype SS17 exhibited on RSE cells.

The evidence found with the EDL933 mutants further supports this observation. EDL933Δ*cah* displayed a decrease in its adherence pattern and less so adherence efficiency to RSE cells compared to wild-type EDL933, indicating it is important but not essential for EDL933's ability to adhere to this cell type. EDL933Δ*iha* showed the same adherence phenotype and aggregation defect in its ability to attach to RSE cells but made EDL933 completely nonadherent to HEp-2 cells. These data suggest that Iha and Cah play a critical role in adherence of EDL933 and other similar strains of O157:H7 to RSE cells, but they are not essential. However, Iha is essential for its ability to adhere to human epithelial cells. What they both are essential for is the aggregative phenotype, as mutations to each gene separately caused a loss in the aggregation phenotype. Both of these data are in opposition to SS17, where deletion of *cah* or *iha* causes no phenotypic difference in adherence to either cell type.

This was especially interesting because in EDL933, *cah* has a paralog and both are full-length genes whereas *cah* in SS17 is missing half of its anchoring *β*-barrel due to the opal frameshift mutation [32, 41]. Despite this, SS17 is still able to confer a stronger adherence to RSE cells than EDL933 with a full-length *cah* (and two copies) that, when deleted, inhibit aggregative adherence of EDL933 to RSE cells, as the adherence phenotype also changes. This would suggest that SS17 has a functionally redundant and perhaps even a more suitable adhesin to adhere to these two cell types, and aggregative and strong adherence on RSE cells independent of *cah* or *iha*.

**Figure 8:**
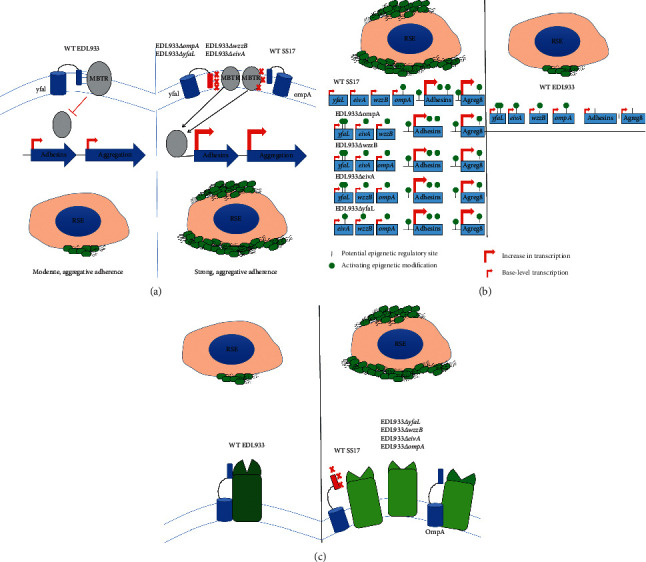
Proposed models driving SS-RSE adherence phenotypes. Based on our data from the EDL933 mutants and the SS17 SNP data, we propose the following models that begin to explain the unique adherence phenotype exhibited by SS isolates on RSE cells. At present, we do not have enough data to narrow down the list of mechanisms, but we propose three possible mechanisms that may be involved in these phenomena. (a) change in interactions with membrane-bound transcriptional regulators (MBTR) such as quorum regulators or *σ*-factors for example. Many global regulators reside in the membranes of *Escherichia coli* and they most likely interact with outer membrane proteins and adhesins. The left panel describes the genetic state of EDL933 adhering to RSE cells where an outer membrane protein (for example, YfaL) can interact with a MBTR. However, in the right panel displaying SS17 with RSE cells, mutations in that transporter (or as is the example with OmpA, mutations with the MBTR) disrupt this regular connection allowing for downstream upregulation of target genes including those involved in adherence to RSE cells and aggregation of the O157:H7 cells. (b) Epigenetic changes can also occur due to changes in the genetic code. In this example, the panel to the left shows the EDL933 mutants and SS17's proposed genome landscape with hypothetical sites of epigenetic regulation. In the case of the right, top panel, in EDL933 parent strain, the epigenetic activity is distributed to express the transporters shown. However, other adhesins and aggregation protein genes may not have preference due to sequence affinity for those enzymes. When SS17 acquired SNPs in these genes or the gene is deleted (as is the case with EDL933Δ*ompA*), the preference switches to lower affinity sites, thus upregulating these other adhesins and aggregation genes. (c) The final model we propose is one that changes the function of neighboring proteins or protein partners of these genes and their gene products. In this example, an autotransporter can interact with another adhesin/aggregation protein in the membrane. This gives the two adhesins a specific yet separate function as they act together. This change in interaction, however, may drive a change in general function and thus a change in phenotype, in this example from a diffuse but strong adherence to a more aggregative and stronger adherence. This change could cause another adhesin or both adhesins to coordinately become stronger and thus drive higher adherence and aggregation, just as it would if these genes were deleted (as in the EDL933 mutants). One alternative to this same hypothesis is a change in another adhesin that we have not tested that interacts with one of the receptors that was tested but does not exhibit an nsSNP (such as OmpA). If the other receptor change caused the dissociation in SS17, a deletion of *ompA* (in this example) would cause the same dissociation in EDL933, causing the change in EDL933's adherence phenotype.

On RSE cells, EDL933Δ*yfaL*, EDL933Δ*ompA*, EDL933Δ*eivA*, and EDL933Δ*wzzB* show a strong and aggregative phenotype whereas the same mutants were either deficient in adherence (Δ*yfaL* and Δ*wzzB*) or had no effect on adherence (Δ*ompA* and Δ*eivA*) to HEp-2 cells. This not only continues to suggest a divergence in adherence mechanisms that seem to be strain and host dependent, but that these genes are involved in the processes involved in the SS adherence phenotype to RSE cells. This is because these mutants and their resulting phenotype on RSE cells are specific to EDL933 (the non-SS isolate); the same mutations have no effect on SS17. The reasons for the differences between the two isolates and their ability to adhere to the different cell types could be caused by any number of mechanisms including change in gene expression caused by interaction with membrane-bound genetic regulatory elements (Figure 8(a)), change in epigenetic regulation of those genes (Figure 8(b)), change in interactions between altered amino acids (Figure 8(c)), or even potentially a combination of a multitude of factors.

SNPs could cause a change in protein function (or protein partner function) or even regulation of other proteins [41]. For example, *yfaL* has three nsSNPs in its effector domain, two of which are nonconservative changes: S23P and D38N. Serine to proline change can cause a kink in the alpha helix portion of the protein that would have been relaxed with serine [47–49]. Serine can also be phosphorylated as a chemical modification, which proline cannot be [50]. This could alter available phosphates in the cell as well as could alter protein-protein interactions that it is involved in.

Another consequence of the SNPs is a change in the overall genetic code, which could influence the function of epigenetic *cis* and *trans* effectors [47]. Among the genes that altered the EDL933 adherence phenotype on RSE cells, three genes exhibited SNPs in SS17 (*wzzB*, *yfaL*, and *eivA*) [41] that could affect the binding of epigenetic effectors. These effectors, in SS17, could then bind to similar sites on other genes that drive the SS phenotype. The EDL933 mutant adherence on RSE cells, such as the *ompA* mutant, could explain another possibility, a change in gene expression. OmpA is ubiquitously expressed in the outer membrane of O157:H7, which is also where a lot of outer membrane transcriptional regulators reside [35]. There could be a change in these regulators, which prevents proper interactions with proteins such as OmpA, and thus, a deletion in *ompA* would cause the SS phenotype in EDL933.

In all, this study has revealed some important new information in determining the mechanisms of adherence to cattle and human epithelial cells. We have determined that adherence of SS17 and EDL933 to RSE and HEp-2 cells is mediated through different factors and mechanisms. These findings, in a small way, show that the factors and mechanisms that underlie adherence and aggregation when O157:H7 EHEC interfaces with different host cells are strain and host cell type dependent. Further studies on other SS strains are needed to determine whether or not these findings can be extrapolated as being a function of SS status.

Because the adherence phenotypes on HEp-2 cells are approximately the same, different factors involved suggest a high level of functional redundancy encoded for by O157:H7 strains and the genes and gene products they use (even if they are different). None of the nonfimbrial genes that were chosen in this study were solely responsible for the strong, aggregative adherence phenotype exhibited by SS17 and the other SS strains that were sequenced for this study. Despite this, the deletion mutants in EDL933 coupled to the results of the HEp-2 adherence experiments brought one gene that should be considered for further analysis, *yfaL*. Deletion of this adhesin resulted in an ablation of adherence to HEp-2 cells for both SS17 and EDL933, but this same deletion resulted in conferring the SS adherence phenotype to EDL933 adhering to RSE cells.

These studies will require further analyses and complementary studies to determine the actual role these genes play in their respective phenotypes, especially for those involved in the SS phenotype (e.g., finding protein partners and epigenetic changes that occur in those genes). Narrowing down these genes could potentially help develop novel therapeutics and better understand the intricate and complex regulatory pathways and interactions involved in adherence of O157:H7 to various cell types. In all, these studies strongly suggest that the initial adherence model for O157:H7 is extremely complex, layered, and strain dependent, more so for the unique adherence phenotypes of SS O157:H7.

## Figures and Tables

**Table 1:** Details of the nonfimbrial genes of interest.

Gene	Amino acid change^a^	Description
*Iha*	—	Fur-regulated adhesin and enterobactin transporter
*ompA*	—	Type 5 secretion system autotransporter
*csgG*	P76 T	Curlin biosynthesis section protein
*wzzB*	P136S	LPS modal length regulator involved in adherence
*eaeH*	T1301 K	Intimin-like homolog protein
*eivA*	P33S	Small protein involved in the biosynthesis of T3SS needle
*yfaL*	S23P D38 N T104I	Type 5 secretion system autotransporter
*Cah*	836 Opal	Type 5 secretion system autotransporter
*Eae*	—	Intimin (adhesin)

^a^Amino acid changes that are specified are in the context of the amino acid positions and are a result of the SNPs in the genes. “—” indicates no amino acid change.

**Table 2:** SNP position and nucleotide change across 9 genes in SS isolate genomes when compared to the EDL933 genome.

O157 isolates	Genes of interest/SNP position										
	*Iha*	*ompA*	*csgG*	*wzzB*	*eaeH*	*eivA*	*yfaL*			*Cah* ^b^	*eae*
			226	403	3902	97	67	112	311	2504	
EDL933	WT^a^	WT	CCG	CCT	ACA	CCC	TCT	GAT	ACC	FL	WT
SS1	WT	WT	CCG	CCT	A**A**A	CCC	TCT	GAT	ACC	FL	WT
SS7	WT	WT	CCG	CCT	A**A**A	CCC	TCT	GAT	ACC	FL	WT
SS12	WT	WT	CCG	CCT	ACA	CCC	TCT	GAT	ACC	FL	WT
SS17	WT	WT	**A**CG	**T**CT	A**A**A	**T**CC	**C**CT	**A**AT	A**T**C	Deletion	WT
SS27	WT	WT	CCG	CCT	A**A**A	CCC	TCT	GAT	ACC	FL	WT
SS42	WT	WT	CCG	CCT	ACA	CCC	TCT	GAT	ACC	FL	WT
SS52	WT	WT	**A**CG	**T**CT	A**A**A	CCC	**C**CT	**A**AT	A**T**C	FL	WT
SS67	WT	WT	**A**CG	CCT	A**A**A	CCC	**C**CT	GAT	ACC	FL	WT
SS77	WT	WT	CCG	CCT	A**A**A	CCC	TCT	GAT	ACC	FL	WT
SS131	WT	WT	CCG	CCT	ACA	CCC	TCT	GAT	ACC	FL	WT
RM11326	WT	WT	CCG	CCT	ACA	CCC	TCT	GAT	ACC	FL	WT
RM11333	WT	WT	CCG	CCT	ACA	CCC	TCT	GAT	ACC	FL	WT

^a^WT: wild type. The *iha* and *ompA* genes do not have previously reported SNPs in SS17. ^b^The *cah* gene contains an opal nonsense mutation in SS17 which is indicated by “deletion”. FL: full length.

## Data Availability

All supporting data can be found in either the supplemental information or by request to the corresponding author.

## Supplementary Materials

Supplementary Materials Table S1: sequencing primers for SS17 adhesins. Table S2: *kanR* cassette generating PCR primers. Table S3: PCR primers for screening single gene inactivation via *kanR* insertion. Table S4: percentages and adherence phenotypes of SS17 and SS17 mutants on HEp-2 and RSE cells. Table S5: percentages and adherence phenotypes of EDL933 and EDL933 mutants on HEp-2 and RSE cells.
