# Supplementary material for: Nonfimbrial Adhesin Mutants Reveal Divergent Escherichia coli O157:H7 Adherence Mechanisms on Human and Cattle Epithelial Cells
Source: Int J Microbiol. 2021 Jan 29;2021:8868151. doi: 10.1155/2021/8868151 (PMC7864753; doi:10.1155/2021/8868151)
Supplement: Supplementary Materials — Table S1: sequencing primers for SS17 adhesins. Table S2: kanR cassette generating PCR primers. Table S3: PCR primers for screening single gene inactivation via kanR insertion. Table S4: percentages and adherence phenotypes of SS17 and SS17 mutants on HEp-2 and RSE cells. Table S5: percentages and adherence phenotypes of EDL933 and EDL933 mutants on HEp-2 and RSE cells. [file 8868151.f1.docx]

**Supplemental Information**

**Table S1. Sequencing primers for SS17 adhesins.**

| **Gene** | **Primer** | **Sequence 5'-3'** |  |
| --- | --- | --- | --- |
| ***csgG*** | csgG-S1A | CCCGCCAGATTTGACATTGC |  |
|  | csgG-S1B | GACAAACCTCGACCATCCAG |  |
| ***wzzB*** | wzzB-S1A | CGTCCCAGAGCAATGTTGTC |  |
|  | wzzB-S1A | CATTGTGGCTATTGCTCTGG |  |
| ***eae*** | eae-S1A | CTCAGAATCAGCGGTACGATG |  |
|  | eaeS2A | CGTTACGTTTCCCTCTCGATG |  |
| ***cah*** | cah-S1A | GAACAGAGGTTGCCGGTATG |  |
|  | cah-S1B | GAACGTGGCGGATCAATGTC |  |
| ***eaeH*** | eaeH-S1A | GACGGCAATGCCAAACAGAC |  |
|  | eaeH-S1B | CGTATAGACACCAGGCTTGC |  |
| ***eivA*** | eivA-S1A | CCAGCATCCGCCTCAAGAAG |  |
|  | eivA-S1B | CAATGCACTGGTGACCCTTC |  |
| ***yfaL*** | yfaL-S1A | CGAAACACGGCGTTAGTGAC |  |
|  | yfaL-S1B | GCTATTGCATCGGGTTGGAC |  |
| ***iha*** | iha-S1A | GTTGCCCTGGAACGTCACTC |  |
|  | iha-S2A | CGGGTCCAGGTGTAATTCAG |  |
|  | iha-S1B | CGAACACGCCATACGGATAG |  |
|  |  |  |  |
|  |  |  |  |
|  | iha-S2B | CTCTCTTCCGTCAATGCAGG |  |
| ***ompA*** | ompA-S1A | GCAGGCATTGCTGGGTAAGG |  |
|  | ompA-S2A | GTCCAGGTCGTCAGTGATTG |  |
|  | ompA-S1B | CCTGACGGAGTTCACACTTG |  |
|  | ompA-S2B | GACCAACAACATCGGTGACG |  |
|  |  |  |  |
|  |  |  |  |

**Table S2. *kanR* cassette generating PCR primers.**

| **Gene** | **Target** | **Sequence 5'-3'** |
| --- | --- | --- |
| ***iha*** | 5' end | gagttgcagtggcaacgtattctaccgtcagtgatagcgttttgttattaTCCCGTCAAGTCAGCGTAAT |
|  | 3' end | tttgtatttgtgtattgtcttgccggttaacatgatcggagattagtaatCAACAAAGCCACGTTGTGTC |
| ***ompA*** | 5' end | caggatctgcaggcattgctgggtaaggaataactgacgaaagtcagttcTCCCGTCAATGTCAGCGTAAT |
|  | 3' end | gcagatcccccggtgaaggatttaaccgtgttatctcgttggagatattcCAACAAAGCCACGTTGTGTC |
| ***csgG*** | 5' end | ggcactcacgctttcgcttaaacagtaaatgccggattattaattccggcTCCCGTCAATGTCAGCGTAAT |
|  | 3' end | cccagcttcataaggaaaataatcatgcagcgcttatttcttttggttgcCAACAAAGCCACGTTGTGTC |
| ***wzzB*** | 5' end | ctgcgtaaacgtcatcagtgagttgcggggtaagcggatgcgatattgttTCCCGTCAAGTCAGCGTAAT |
|  | 3' end | ttatcctatagcattcacagggattatcgctaaactatgcggacttggaaCAACAAAGCCACGTTGTGTC |
| ***cah*** | 5' end | tctcttgcgtgactgctctactgttaatagaataaaacgatcgataaaacCAACAAAGCCACGTTGTGTC |
|  | 3' end | accgtcatcatccttaacatcaacggaagaatggcctgcagcaccatacaTCCCGTCAAGTCAGCGTAAT |
| ***eaeH*** | 5' end | tatttgtgtctgcctatgttcgttaattcgttcatcaggaaattatctcaCAACAAAGCCACGTTGTGTC |
|  | 3' end | tcaacgccattattgtttattagaatgttacttccatattcttaatattaTCCCGTCAAGTCAGCGTAAT |
| ***eivA*** | 5' end | ataattttaattttttcataccagcatgctccatatcgcaagacattatcTCCCGTCAAGTCAGCGTAAT |
|  | 3' end | acaaaaaacaaaataaaaaaccatggaagaaggtgatgttcaatgtttaaCAACAAAGCCACGTTGTGTC |
| ***yfaL*** | 5' end | ctgattaggcgtttacgccgcatccagcaatagtgatggtcagacagcgaTCCCGTCAAGTCAGCGTAAT |
|  | 3' end | tataatgcgtttaaatatgccgtcttatataaaatgatggtcagattaatCAACAAAGCCACGTTGTGTC |
| ***eae*** | 5' end | ttggtatcagcgtggttggatcaacctacatgagaaaacgtgaatgtgtcTCCCGTCAAGTCAGCGTAAT |
|  | 3' end | atccgatctattaatataatttatttctcattctaactcattgtggtggaCAACAAAGCCACGTTGTGTC |
|  |  |  |
|  |  |  |

**Table S3. PCR primers for screening single gene inactivation via *kanR* insertion.**

| **Gene** | **Primer** | **Sequence 5'-3'** |
| --- | --- | --- |
| ***iha*** | iha-A | GGTGGTGACCCTGCATTCTG |
|  | iha-B | GGAGCATGTCAGGCAAGACG |
| ***ompA*** | ompA-A | CGATGTGTTGCTCTCGCTGG |
|  | ompA-B | CGGGAATGGGTTCAGGCATC |
| ***csgG*** | csgG-A | CTGCTTAGTGGCGTGGTGTG |
|  | csgG-B | GACAGCTCTCTTGCAGCACC |
| ***wzzB*** | wzzB-A | GCCGCGACTGTACATGACCG |
|  | wzzB-A | TCTCTTTGGCAGCGACTAAC |
| ***cah*** | cah-A | GCAGGTATTCAGCCTTCTCC |
|  | cah-B | GAACGTGGCGGATCAATGTC |
| ***eaeH*** | eaeH-A | GCGATGGGTAGTGCAAGTTC |
|  | eaeH-B | CTATGAAGGTGAGTGGGAGC |
| ***eivA*** | eivA-A | CCCTACCACGTTCGCCAATC |
|  | eivA-B | TGTCCGGCTAAACGCTGAAG |
| ***yfaL*** | yfaL-A | GGGCAGTATGATGTGGTGAC |
|  | yfaL-B | TGAACGGCAGAATAGGTTGG |
| ***eae*** | eae-A | CTCAGAATCAGCGGTACGATG |
|  | eae-B | CGTTACGTTTCCCTCTCGATG |
| ***KanR*** | KanR-C | GTATTTCGTCTCGCTCAGGC |
|  | KanR-D | GATGTTGGACGAGTCGGAAT |
|  | KanR-C2 | GCCTGAGCGAGACGAAATAC |
|  | KanR-D2 | GATGTTGGACGAGTCGGAAT |

**Table S4. Percentages and Adherence Phenotypes of SS17 and SS17 Mutants on Hep-2 and RSE cells.**

|  |  |  | Adherent Bacteria | | | SEM | | | |  | |  | |  | |  |
| --- | --- | --- | --- | --- | --- | --- | --- | --- | --- | --- | --- | --- | --- | --- | --- | --- |
|  |  |  | > 10 per cell | | <10  per cell | | > 10 per cell | | <10 per cell | |  | |  | |  | |
| **SS17** | **Wild Type** | RSE | 95 | 2.5 | | 5 | | 2.5 | |  | |  | |  | |  |
|  |  | HEp-2 | 0 | 60.5 | | 0 | | 0.5 | |  | |  | | Aggregative, Strong | |  |
|  | **Δ*yfaL*** | RSE | 98.75 | 1.25 | | 1.25 | | 1.25 | |  | |  | | Aggregative, Moderate | |  |
|  |  | HEp-2 | 0 | 30 | | 0 | | 5 | |  | |  | | Diffuse, Strong | |  |
|  | **Δ*eivA*** | RSE | 97 | 3 | | 3 | | 3 | |  | |  | | Diffuse, Moderate | |  |
|  |  | HEp-2 | 0 | 57.5 | | 0 | | 4.5 | |  | |  | | Non-Adherent | |  |
|  | **Δ*eaeH*** | RSE | 100 | 0 | | 0 | | 0 | |  | |  | |  | |  |
|  |  | HEp-2 | 0 | 46 | | 0 | | 3.25 | |  | |  | |  | |  |
|  | **Δ*ompA*** | RSE | 87.1 | 13.25 | | 1 | | 0.75 | |  | |  | |  | |  |
|  |  | HEp-2 | 12.5 | 55.25 | | 1.25 | | 6.25 | |  | |  | |  | |  |
|  | **Δ*cah*** | RSE | 87.5 | 12.5 | | 12.5 | | 12.5 | |  | |  | |  | |  |
|  |  | HEp-2 | 6.5 | 72 | | 6.5 | | 2 | |  | |  | |  | |  |
|  | **Δ*iha*** | RSE | 97 | 3 | | 6.5 | | 6.5 | |  | |  | |  | |  |
|  |  | HEp-2 | 0 | 82.5 | | 0 | | 29.5 | |  | |  | |  | |  |
|  | **Δ*wzzB*** | RSE | 90 | 10 | | 3 | | 3 | |  | |  | |  | |  |
|  |  | HEp-2 | 0 | 67.5 | | 0 | | 8.5 | |  | |  | |  | |  |
|  | **ΔcsgG** | RSE | 94.5 | 3.5 | | 10 | | 10 | |  | |  | |  | |  |
|  |  | HEp-2 | 0 | 59.5 | | 0 | | 6.5 | |  | |  | |  | |  |
|  | **Δ*eae*** | RSE | 87.5 | 12.5 | | 1.25 | | 1.25 | |  | |  | |  | |  |
|  |  | HEp-2 | 0 | 29.5 | | 0 | | 6.5 | |  | |  | |  | |  |

**Table S5. Percentages and Adherence Phenotypes of EDL933 and EDL933 Mutants on Hep-2 and RSE cells.**

|  |  |  | Adherent Bacteria | | SEM | | |  | | |  | | |  | | |  |
| --- | --- | --- | --- | --- | --- | --- | --- | --- | --- | --- | --- | --- | --- | --- | --- | --- | --- |
|  |  |  | > 10  per cell | <10  per cell | | > 10  per cell | <10  per cell | |  |  | | |  | | |  |  |
| **EDL933** | **Wild Type** | RSE | 41 | 59 | | 7 | 7 | |  |  | | |  | | |  |  |
|  |  | HEp-2 | 12 | 61.5 | | 12 | 6.5 | |  |  | | | Aggregative, Strong | | |  |  |
|  | **Δ*yfaL*** | RSE | 81 | 18.5 | | 2.5 | 2.5 | |  |  | | | Aggregative, Moderate | | |  |  |
|  |  | HEp-2 | 0 | 33 | | 0 | 2 | |  |  | | | Diffuse, Strong | | |  |  |
|  | **Δ*eivA*** | RSE | 79.25 | 20.75 | | 1.75 | 1.75 | |  |  | | | Diffuse, Moderate | | |  |  |
|  |  | HEp-2 | 0 | 74 | | 0 | 2 | |  |  | | | Non-Adherent | | |  |  |
|  | **Δ*eaeH*** | RSE | 61.25 | 38.75 | | 8.75 | 7.5 | |  |  | | |  | | |  |  |
|  |  | HEp-2 | 0 | 91 | | 0 | 1 | |  |  | | |  | | |  |  |
|  | **Δ*ompA*** | RSE | 100 | 0 | | 0 | 0 | |  |  | | |  | | |  |  |
|  |  | HEp-2 | 0 | 57.5 | | 0 | 7.5 | |  | | |  | | |  | | |
|  | **Δ*cah*** | RSE | 7.5 | 93 | | 7.5 | 7 | |  | | |  | | |  | | |
|  |  | HEp-2 | 0 | 82.5 | | 0 | 7.5 | |  | | |  | | |  | | |
|  | **Δ*iha*** | RSE | 38 | 62 | | 2 | 2 | |  | | |  | | |  | | |
|  |  | HEp-2 | 0 | 45.5 | | 0 | 1.5 | |  | | |  | | |  | | |
|  | **Δ*wzzB*** | RSE | 83.5 | 16.5 | | 2.5 | 2.5 | |  | | |  | | |  | | |
|  |  | HEp-2 | 0 | 23 | | 0 | 5 | |  | | |  | | |  | | |
|  | **ΔcsgG** | RSE | 49.5 | 50.5 | | 6.5 | 6.5 | |  | | |  | | |  | | |
|  |  | HEp-2 | 0 | 95 | | 0 | 5 | |  | | |  | | |  | | |
